# The Effect of Smartphone Apps Versus Supervised Exercise on Physical Activity, Cardiorespiratory Fitness, and Body Composition Among Individuals With Mild-to-Moderate Mobility Disability: Randomized Controlled Trial

**DOI:** 10.2196/14615

**Published:** 2020-02-04

**Authors:** Daniel Berglind, Diego Yacaman-Mendez, Catharina Lavebratt, Yvonne Forsell

**Affiliations:** 1 Department of Global Public Health Karolinska Institutet Stockholm Sweden; 2 Department of Molecular Medicine and Surgery Karolinska Institutet Stockholm Sweden

**Keywords:** mobility disability, physical activity, cardiorespiratory fitness, exercise, randomized controlled trial, app, smartphone

## Abstract

**Background:**

Adequate levels of physical activity (PA) and good cardiorespiratory fitness (CRF) are associated with profound health benefits for individuals with mobility disability (MD). Despite the vast amount of research published in the field of PA interventions, little attention has been given to individuals with MD.

**Objective:**

The aim of this study was to examine the efficacy of an app-based versus a supervised exercise and health coaching program to support adults with MD to increase levels of PA, CRF, and improve body composition.

**Methods:**

Participants with self-perceived MD, aged 18 to 45 years, were included in this 12-week parallel-group randomized controlled trial and allocated at random to an app-based intervention, using commercially available apps—the Swedish Military training app (FMTK), the Acupedo walking app, and the LogMyFood food photography app—or a supervised exercise and health coaching intervention, including 1 weekly supervised exercise session and healthy lifestyle coaching. The primary outcome was the level of moderate-to-vigorous PA (MVPA) measured with accelerometers. Secondary outcomes included CRF measured by a submaximal test performed on a stationary bicycle and body composition measured by bioelectrical impedance. All outcomes were measured at baseline, 6 weeks, and 12 weeks. Linear mixed-effect models were used to assess the between-group differences, as well as the within-group changes through time, in each intervention group.

**Results:**

A total of 110 participants with MD were randomized to an app-based intervention (n=55) or a supervised exercise and health intervention (n=55). The mean age of participants was 34.9 years (SD 6.1), and 81.8% (90/110) of the participants were women. CRF showed a moderate increase in both groups after 12 weeks—1.07 (95% CI –0.14 to 2.27) mL/kg/min increase in the app-based group and 1.76 (95% CI 0.70 to 2.83) mLkg/min increase in the supervised exercise group. However, the intention-to-treat analysis showed no significant differences between the groups in MVPA or CRF after 12 weeks. Waist circumference was significantly lower in the app-based intervention group.

**Conclusions:**

Commercially available apps increased levels of CRF and improved body composition over 12 weeks to the same extent as supervised exercise sessions, showing that both are equally effective. However, neither the app-based intervention nor the supervised exercise intervention increased MVPA.

**Trial Registration:**

International Standard Randomized Controlled Trial Number (ISRCTN): 22387524; http://isrctn.com/ISRCTN22387524.

## Introduction

### Background

In Sweden, approximately 23% of the population, between 16 and 84 years of age, reports some kind of disability, whereas 8% of the population suffers from mobility disability (MD), limiting their participation in society and ability to work. Health-related and social welfare costs for individuals with disabilities are the fastest growing expenditure by municipality in Sweden, and this represents 1.6% of the Swedish gross domestic product [1]. Often, individuals with MD suffer from social and health inequalities related to their condition, such as impaired access to health care services or social programs, lower employment rates, and higher risk of chronic diseases [2-4].

Although there are many known health benefits of physical activity (PA) and cardiorespiratory fitness (CRF), such as reduced risk for cardiovascular disease and improvement in markers of metabolic health [5,6], young adults with MD are less likely to engage in PA compared with their able-bodied peers [7,8]. In addition, obesity has been shown to be both a risk factor and a consequence of MD [9,10], and observational studies have shown that individuals with MD are not only more likely to suffer from obesity, but they are also more likely to be negatively impacted by its consequences [10].

Despite the vast amount of research published in the field of PA interventions over the past decades, little attention has been given to interventions aiming to increase PA and CRF among individuals with MD. Nevertheless, interventions focusing on PA and CRF for those with MD may be of particular importance, as there is evidence indicating that PA and CRF can confer additional health benefits for people with MD compared with the general population [11]. Additional evidence for the importance of high CRF in young adulthood comes from a recent prospective study, showing that higher CRF in young adulthood is associated with lower risk for receipt of a disability pension (from, eg, musculoskeletal causes) later in life, in a dose-response manner across all BMI categories [12]. To date, PA interventions targeting individuals with MD are limited to labor-intense supervised health programs targeting the elderly [13].

The few existing studies on PA and MD indicate that motivation for PA is high among individuals with MD and that barriers to PA engagement primarily include accessibility to tailored PA and a lack of knowledge on how to engage in PA [7,14]. Moreover, there is evidence that autonomy, goal setting, surveillance, support, and feedback are important factors for improving and maintaining healthy levels of PA in young adults with MD [14]. Consequently, factors beyond health benefits may be important to target when intervening on levels of PA and CRF in individuals with MD. Given that many people have busy lifestyles but still value access to health behavior programs that provide advice, information, feedback, and self-monitoring around the clock, app-based programs may be an attractive approach [15]. A recent meta-analysis showed modest evidence for effectiveness of smartphone apps to increase PA in the short term (up to 3 months) [16]. However, there is an inconsistency in the literature on the effects from multicomponent versus app-based PA interventions on health outcomes [17]. A preventive app-based PA intervention targeting individuals with MD has the potential to improve levels of PA, CRF, and the general health in this vulnerable group, to avert the progression to more severe disabilities and comorbidities and to thus reduce social and health care inequalities.

### Objectives

The aim of this randomized controlled trial (RCT) was to evaluate the effects of commercially available apps compared with a supervised exercise and health program on levels of PA (primary outcome), CRF, and body composition.

## Methods

### Trial Design

The parallel-group RCT study presented in this paper was designed to examine the effects of an app-based program compared with a supervised exercise and health program on levels of PA. Secondary outcomes included CRF and body composition. The trial has been approved by the Ethical Review Board Stockholm (Dnr: 2017/1206-31/1) and registered in the ISRCTN registry (registration number: ISRCTN22387524) [18]. A detailed description of this trial has been published previously [19].

### Participants

Participants were recruited from rehabilitation and primary care centers and from occupational health care within the Stockholm (Sweden) area. Recruitment started in May 2018, and the last 12-week measurements were finished in December 2018. All participants gave written consent before entering the trial. The eligibility criteria comprised the following: both sexes aged 18 to 45 years, who reported having experienced any mobility-related problems affecting their everyday life, for example, problems with dressing, performing household tasks, at transportation, personal hygiene tasks, or at work in the past 3 years before enrollment in the trial. Participants who were bound to a wheelchair or whose medical condition prevented them from moderate-intensity walking, as well as people unable to speak and read Swedish or who did not have access to a smartphone, were excluded.

### Randomization

Group assignment was randomly generated, after the baseline measurements, via a block randomization procedure (in blocks of 2 to ensure an equal distribution of participants between the 2 treatment groups), using the SAS Proc Plan (SAS Institute Inc).

### Blinding

This was an investigator-blinded study. The nature of the activities in the groups made blinding the participants unmanageable. The assessment staff members were blinded to the intervention and remained separate from the intervention team. Participants were asked not to disclose their assigned group during the assessments.

### Interventions

The treatment arms in the trial were designed with an intrinsic motivation strategy behavior change theory framework [20] to support participants to perform sustained changes in moderate-to-vigorous PA (MVPA) and CRF.

### The App-Based Program

The app-based program was a 12-week walking and exercise program, delivered via commercially available smartphone apps, aiming to engage participants in at least 30 min of daily MVPA. Apps used were the Acupedo walking app, with inbuilt goal setting and feedback options, an individually tailored home-based bodyweight exercise app developed by the Swedish Military (FMTK), and the LogMyFood food photography app. Both the Acupedo and the FMTK apps encourage PA that can be detected as MVPA by hip-worn accelerometers. The intervention further included 3 face-to-face consultations, in groups of approximately 20 participants, where information on how to use the apps (session 1 at baseline), goal settings (session 2 at 6 weeks), and motivation to continue exercise (session 3 at 12 weeks) were discussed. At the 12-week follow-up, participants reported how often they had used the apps throughout the intervention (intervention adherence).

### The Supervised Health Program

The supervised health program was based on the transtheoretical and sociocognitive models of behavior change [21]. It was a 12-week standard care exercise and health coaching program, delivered by health educators and personal trainers, including 1 weekly exercise session supervised by a personal trainer, with aerobic and strength exercises (a total of 12 sessions) and 3 meetings (baseline, week 6 and 12) with a health educator/dietitian. The personal meetings were based on a behavior change model with 4 core behavior change techniques (mobilizing social support for change, developing self-efficacy, goal setting, and self-monitoring), which are known to be effective in supporting individuals to improve healthy activity and dietary behaviors [22]. Dietary advice given to the participants followed the 4-step Step-wise Weight-determined Accumulative change Plan model [23].

PA goals and exercise programs were individualized and modified in response to baseline levels of CRF (VO_2_max), illness, injury, or physical symptoms, in collaboration with the personal trainer. Each personal trainer session also included a short (5-10 min) motivation/feedback part. Participants were moreover encouraged to have an active lifestyle with at least 2 more weekly nonsupervised exercise sessions and to engage in a minimum of 30 min of daily MVPA.

### Measurements

Measures on all outcomes were taken at baseline (week 0), midpoint (week 6), and at the end of the intervention (week 12). A Web-based survey was used to collect all questionnaire data. In addition to all outcome measures, the baseline assessment further included self-reported demographic and contact information, medical history, and living habits.

### Primary Outcome

The primary outcome, between-group differences in minutes spent in MVPA per day at 12 weeks, was measured objectively, using Actigraph GT3X+ accelerometers, worn on the hip during all waking hours, for 7 consecutive days at each assessment. Sedentary time and light PA (LPA) were also measured by the accelerometer. Management and analyses of PA data followed best-practice and research recommendations [24]. Valid measurements included ≥10 hours wear time per day for ≥4 days. Vector magnitude (√X^2^+Y^2^+Z^2^) was analyzed and recorded in 10-second epochs, converted to counts per minute (cpm). Wear time and classification of bouts were computed using ActiLife v.6.13.3, using an algorithm by Choi et al [25], and nonwear time was classified as nonzero counts for at least 60 min, with a maximum break of 2 min. We classified MVPA as more than 3208 cpm [26].

### Secondary Outcomes

#### Cardiorespiratory Fitness

CRF was measured via a submaximal VO_2_max test, performed on a stationary bicycle, according to the Ekblom-Bak cycle ergometer test [27], and presented in relative numbers as mL/kg/min.

#### Body Composition

Fat mass (kg) and fat-free mass (kg) were assessed via bioelectrical impedance [28], using an Omron model HBF-511B-E/HBF-511T-E. The physical tests further included height, weight, and waist circumference, measured by validated instruments, with the participants wearing light clothes to the nearest 0.1 kg and 0.5 cm, respectively.

#### Power Calculation

Power calculations for the primary outcome, between-group differences in minutes spent in MVPA per day at 12 weeks, were based on a 2-sided log-rank test at the 5% significance level. Under these assumptions, randomization of 80 individuals (40 individuals per group) provides 80% power to detect a between-group difference of 10 min of daily MVPA.

### Statistical Analyses

Participant’s baseline clinical and demographic characteristics are presented in Table 1, using frequencies and percentages for binary variables and mean and SD for continuous data.

Linear mixed-effect models (LMM) with a random intercept were fitted to estimate the between- and within-group differences for the main and secondary outcomes using time, group allocation, and their interaction as explanatory variables, adjusting for sex, and baseline VO_2_max and BMI values as possible confounders in all models. Correlations because of paired data were modeled using an unstructured covariance matrix. To estimate the pairwise changes in each of the groups, correction for multiple tests was performed using the Bonferroni method.

**Table 1:** Baseline characteristics of the participants by randomization group.

Characteristics		App group (n=55)		Supervised exercise group (n=55)	
Age (years), mean (SD)		35.6 (6.2)		34.5 (6.5)	
Women, n (%)		47 (85)		43 (78)	
**Smoking, n (%)**					
	Daily smoking		3 (5)		2 (3)
	Smoking occasionally		11 (20)		5 (9)
**Education, n (%)**					
	Elementary school		3 (5)		2 (3)
	High school		12 (21)		14 (25)
	University		37 (67)		36 (65)
Moderate-to-vigorous physical activity (min/day), mean (SD)		48.4 (23.3)		40.3 (20.6)	
**Cardiorespiratory fitness, mean (SD)**					
	VO_2_max, mL/kg/min		36.0 (7.9)		35.3 (8.7)
**Body measures, mean (SD)**					
	Weight, kg		77.6 (20.4)		77.7 (17.3)
	Height, cm		171.8 (9.4)		171.0 (9.3)
	BMI, kg/m^2^		26.3 (5.7)		27.2 (5.2)
	Fat mass, kg		27.1 (12.5)		28.3 (11.9)
	Fat-free mass, kg		49.1 (8.4)		51.4 (10.6)
	Waist circumference, cm		86.9 (18.3)		84.6 (11.7)

No imputation of missing data was performed, and the mechanism of missing data is assumed to be at random, that is, missing data are not dependent on unobserved confounders. Given that LMM uses all the available information at baseline to calculate individual effects, estimations are also made for those lost to follow-up [29].

For the primary analysis, intention-to-treat analysis was conducted [19], on individuals according to group randomization [30]. We also ran per-protocol analyses, including those in the app group, who used the apps ≥5 days per week, and those in the supervised exercise group, who attended ≥10 exercise sessions. Further sensitivity analyses included comparisons of baseline characteristics of participants lost to follow-up by randomization group and by dropouts.

All statistical tests were calculated using a 2-tailed .05 significance level.

BMI was calculated as weight (kg)/height (m)^2^. For MVPA and CRF, within- and between-group trajectories (from baseline to week 6 and 12) were calculated from a repeated measure mixed-effects model (time × group interactions) to explore the differences in MVPA and CRF at each time point between the groups. Change variables were created for between-group comparisons on changes in the primary and secondary outcomes from baseline to 6 and 12 weeks, respectively. All statistical analyses were performed using Stata/IC 15.1.

### Consent for Publication

Published data will not contain any personal identification numbers. Thus, no single individual participating in the trial can be identified by published results.

## Results

### Study Participants

**Figure 1:**
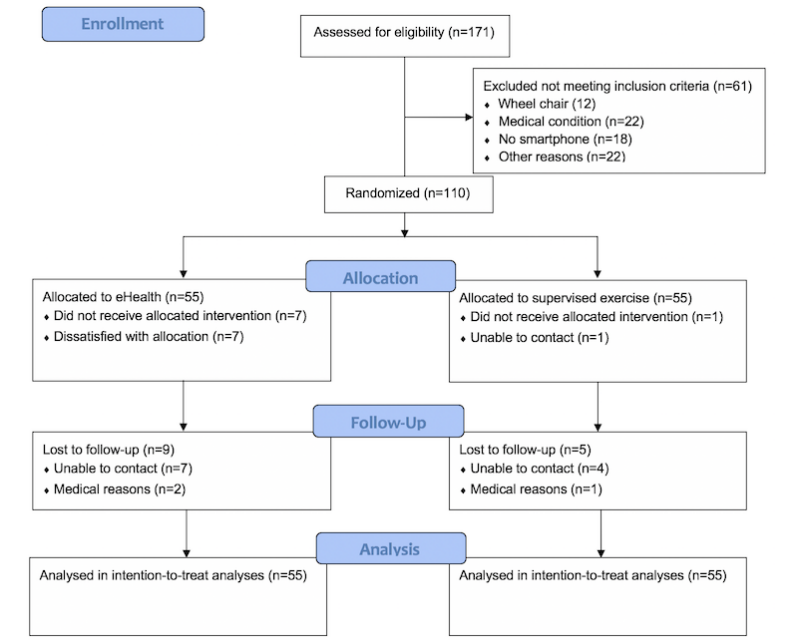
Flow chart according to Consolidated Standards for Reporting of Trials. eHealth: electronic health.

A participant flow diagram is shown in Figure 1. In total, 171 individuals were assessed for eligibility, of whom 61 were excluded because of being bound to a walker or wheelchair (n=12), having any medical condition not permitting moderate-intensity walking (n=22), not having access to a smartphone (n=18), or other reasons (n=10).

### Intervention Adherence

A total of 36% (20/55) participants in the app group used the apps daily, 31% (17/55) for 5 to 6 days/week, 14% (8/55) for 3 to 4 days/week, and 19% (10/55) for less than 3 times/week. In the supervised exercise group, 45% (25/55) of the participants attended all 12 personal trainer exercise sessions, 29% (16/55) attended 11 exercise sessions, 7% (4/55) attended 10 exercise sessions, and the remaining 19% (10/55) attended 4 to 9 exercise sessions.

### Participant’s Baseline Characteristics

Baseline characteristics of the participants by randomization group are shown in Table 1. No differences were found between the groups with regard to baseline characteristics. A majority of the participants met the daily recommended minimal PA guideline of ≥150 min of MVPA/week [31]. Accelerometer wear time did not differ between the groups, 14.5 (SD 1.3) h/day and 14.1 (SD 1.1) h/day in the app and supervised exercise group, respectively. All the included participants reported mobility-related problems affecting their everyday life, and 88.2% of the participants reported a chronic illness defined as “problems causing work ability to be impaired or hindering other daily lives pursuits.”

### Between-Group Differences at 6 and 12 weeks

**Figure 2:**
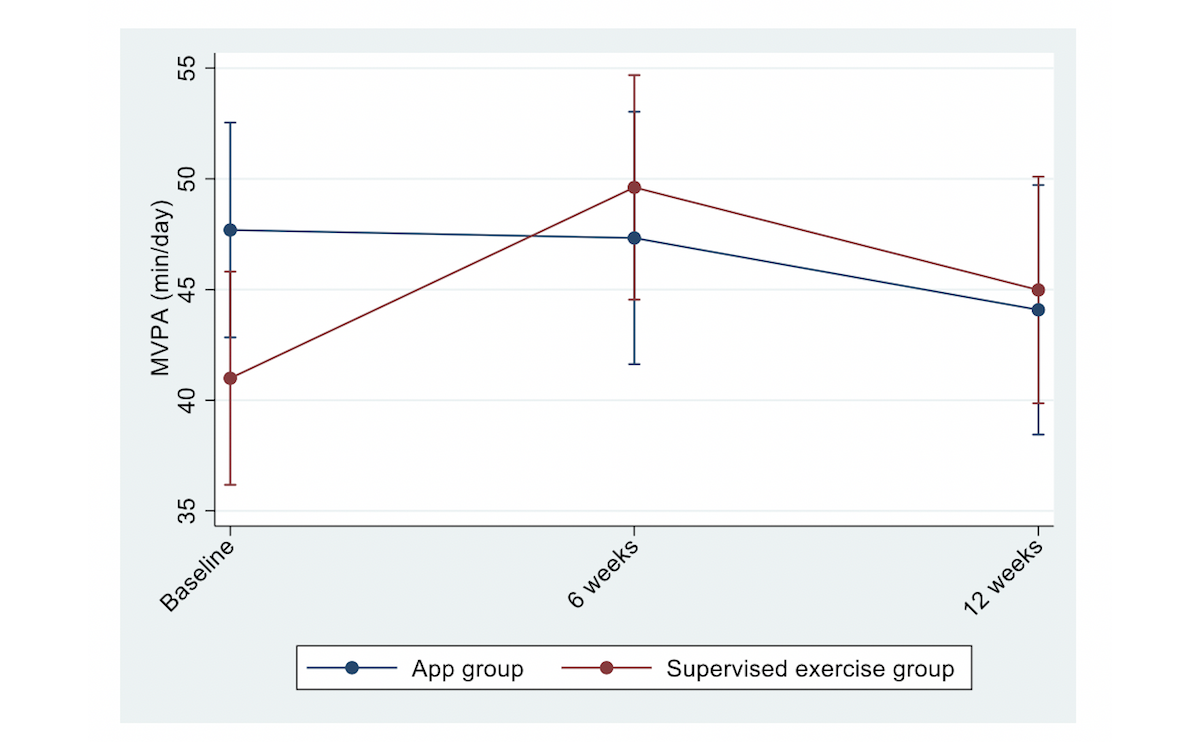
Levels of moderate-to-vigorous physical activity at each time point between the groups. Adjusted for sex, BMI, and maximal oxygen uptake (VO_2_max). MVPA: moderate-to-vigorous physical activity.

**Figure 3:**
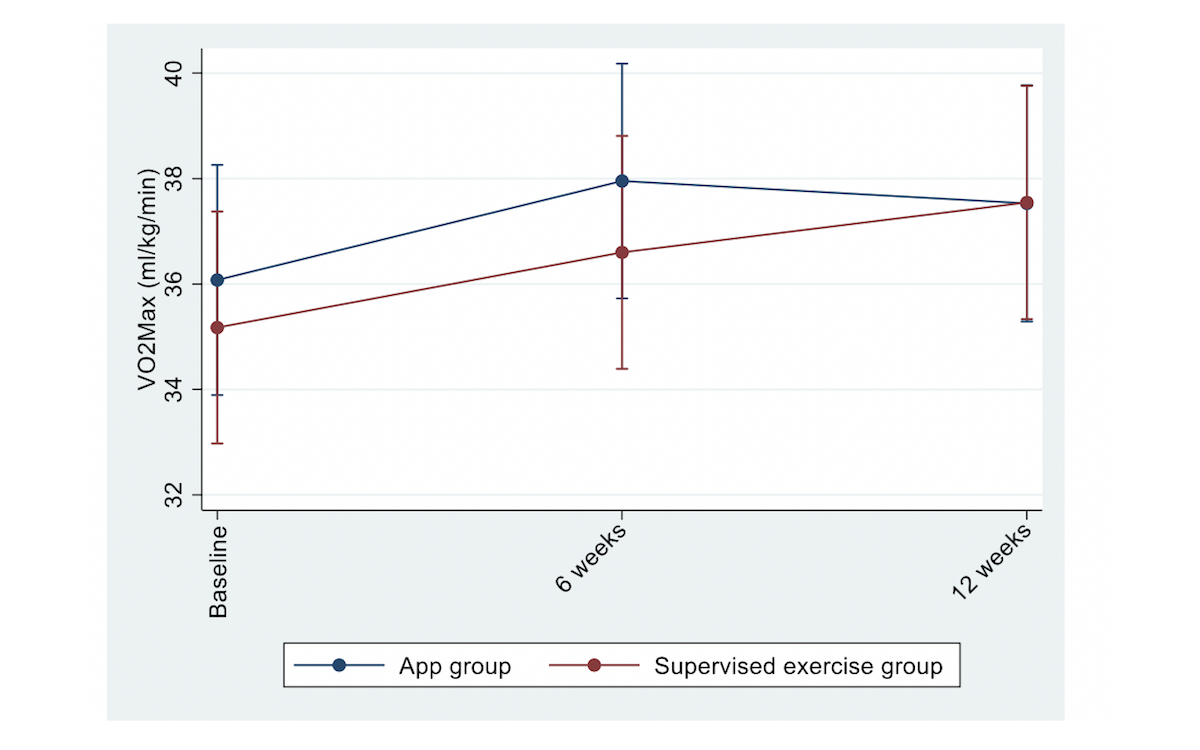
Levels of cardiorespiratory fitness (VO_2_max) at each time point between the groups. Adjusted for sex, BMI, and VO_2_max. MVPA: moderate-to-vigorous physical activity. VO_2_max: maximal oxygen uptake.

Between-group differences at 6 and 12 weeks in the primary outcome (MVPA) and secondary outcomes are shown in Figures 2 and 3 and in Table 2. No significant differences, except for waist circumference, were found between the groups for any of the outcomes at 12 weeks. Accelerometer wear time did not differ between the groups at 6 weeks—14.3 (SD 1.5) h/day and 14.4 (SD 1.4) h/day in the app and supervised exercise group, respectively—and 12 weeks—14.6 (SD 1.5) h/day and 14.5 (SD 1.4) h/day in the app and supervised exercise group, respectively. A total of 90% (41/45) and 92% (46/50) of participants in the app and supervised exercise group, respectively, met the PA recommendations at 12 weeks.

**Table 2:** Results of the intention-to-treat analysis. Mean difference of primary and secondary outcomes between the app group and the supervised exercise group, as well as within each treatment group (adjusted for sex, body mass index, and VO_2_max; pairwise comparisons are adjusted using Bonferroni correction).

Outcomes	Between-group differences^a^, beta coefficient, Δ (0 to 12 weeks)	Within-group differences			
		App group		Supervised exercise group	
		Beta coefficient, Δ (0 to 6 weeks)	Beta coefficient, Δ (0 to 12 weeks)	Beta coefficient, Δ (0 to 6 weeks)	Beta coefficient, Δ (0 to 12 weeks)
Moderate-to-vigorous physical activity, min/day	–5.71 (–12.91 to 1.47)	–2.25 (–10.40 to 5.90)	–6.17 (–14.28 to 1.95)	6.60 (–0.74 to 13.90)	1.37 (–6.06 to 8.81)
VO_2_max, mL/kg/min	–0.53 (–2.40 to 1.34)	*1.32 (0.13 to 2.52)* ^b^	1.07 (–0.14 to 2.27)	1.00 (–0.07 to 2.07)	*1.76 (0.70 to 2.83)* ^c^
Weight, kg	–2.17 (–4.75 to 0.42)	0.15 (–0.35 to 0.66)	–0.03 (–0.53 to 0.47)	0.28 (–0.17 to 0.72)	–0.02 (–0.49 to 0.45)
BMI, kg/m^2^	0.86 (–1.02 to 2.73)	–0.09 (–0.43 to 0.27)	–0.19 (–0.54 to 0.16)	–0.16 (–0.46 to 0.15)	–*0.40 (–0.72 to* *–0.07)*^d^
Fat mass, kg	–0.86 (–2.00 to 0.28)	–0.01 (–0.90 to 0.92)	0.20 (–0.72 to 1.20)	0.32 (–0.50 to 1.13)	*0.92 (0.09 to 1.75)* ^b^
Fat-free mass, kg	–0.34 (–2.67 to 1.99)	0.77 (–0.65 to 2.19)	0.52 (–0.93 to 1.96)	–0.08 (–1.35 to 1.20)	–1.24 (–2.54 to 0.06)
Waist circumference, cm	–*4.27 (–7.78 to* –*0.76)*^b^	–0.16 (–1.90 to 1.57)	–0.75 (–2.49 to 0.99)	1.10 (–0.44 to 2.64)	–0.76 (–2.34 to 0.82)

^a^Reference: supervised exercise group.

^b^*P*<.05.

^c^*P*<.001.

^d^*P*<.01.

For the main outcome of MVPA, there was a nonsignificant reduction after 12 weeks of intervention, and no significant difference between the groups was observed. With respect to the secondary outcomes, there was a significant increase in CRF in both groups, but no significant difference between the treatment groups.

### Changes in Outcomes From Baseline to 6 and 12 Weeks Within the Groups

Pairwise comparison of the changes in primary and secondary outcomes at 6 weeks and 12 weeks by randomization group are shown in Table 2. Participants in the supervised exercise group increased their levels of MVPA by 8%, whereas participants in the app group decreased their MVPA by 9%. VO_2_max increased significantly, from baseline to 12 weeks, in both groups, 4% in the app group and 7% in the supervised exercise group. In the app group, 33 (33/38, 87%) participants met the PA recommendations at both baseline and 12 weeks, 2 (2/38, 5%) participants went from not meeting to meeting the PA recommendations, and 3 (3/38, 8%) participants went from meeting to not meeting the PA recommendations. In the supervised exercise group, 42 (42/50, 84%) participants met the PA recommendations at both baseline and 12 weeks, 6 (6/50, 12%) participants went from not meeting to meeting the PA recommendations, and 2 (2/50, 4%) participants went from meeting to not meeting the PA recommendations. Both groups showed decreases in weight and waist circumference from baseline to 12 weeks. However, only participants in the app group decreased their fat-free mass from baseline to 12 weeks.

### Per-Protocol Analyses

Per-protocol analyses included those in the app group, who used the apps ≥5 days per week (20/39, 51%), and those in the supervised exercise group, who attended ≥10 exercise sessions (45/49, 92%). These analyses showed that those in the app group had a significantly higher VO_2_max and lower BMI, waist circumference, and fat-mass compared with those in the supervised exercise group at 6 and 12 weeks. However, participants in the app group did not show any significant improvements from baseline to 6 and 12 weeks for any of the measured variables (Multimedia Appendix 1).

### Sensitivity Analyses

Between-group differences from baseline to 12 weeks in the primary and secondary outcomes, including participants with complete data at 12 weeks (n=39 for the app group and n=49 for the supervised exercise group), showed similar results as the intention-to-treat analysis (Multimedia Appendix 1). There were no between-group differences in baseline characteristics of participants lost to follow-up by randomization group (Multimedia Appendix 1) and by dropouts (Multimedia Appendix 1).

### Adverse Events

A total of 2 participants (1 in the app group and 1 in the supervised exercise group) cancelled participation in the trial because of illness. However, none of these illnesses were related to the intervention.

## Discussion

### Principal Findings

This single-centered RCT aimed to compare the effect of an app-based program and supervised exercise and health program among people with mild-to-moderate MD. We found a moderate increase in CRF after 12 weeks of follow-up in both groups. There was no significant difference between groups in our main outcome, time spent in MVPA, nor in CRF measured by VO_2_max or most measurements of body composition (weight, BMI, fat mass, and fat-free mass). Participants randomized to the app-based intervention had a significantly lower waist circumference after 12 weeks of follow-up in the intention-to-treat analysis. However, the per-protocol analysis showed a significant difference in CRF and BMI between groups. The app-based intervention group had a lower BMI and higher CRF after 12 weeks of follow-up. Thus, a health program using commercially available health apps is a feasible intervention to improve health among people with mild-to-moderate MD.

### Comparison With Previous Work

Current systematic review data indicate that app-based interventions to improve PA can be effective and that multicomponent interventions appear to be more effective than stand-alone app interventions [17]. However, there is a lack of randomized trials, and most previous studies have included older populations [32] or relied on self-reported measures of PA [17]. The discrepancy between self-reported and objectively measured PA is well established [33], and several app-based PA intervention studies show substantial increases in self-reported PA but not objectively (accelerometer) measured PA [34,35]. This lack of uniformity, in combination with the inaccuracy of self-reported data as a measure of PA, makes it difficult to compare and summarize outcomes across PA interventions.

PA interventions, using objective measures of PA, targeting individuals with MD are scarce. The LIFE intervention randomized 1635 sedentary 70- to 89-year-old men and women with MD to an exercise program, with supervised exercise 2 times per week and home-based exercise 3 to 4 times per week, or to a health educator program comprising workshops on topics relevant to older adults [13]. Through 2 years of follow-up, participants in the exercise group participated in an additional 40 min of light-intensity PA per week, assessed by accelerometry, compared with the health educator group. The between-group differences in self-reported PA were 104 min per week. This discrepancy between self-reported and objectively measured PA further highlights the importance to incorporate objective measures when evaluating the effectiveness of PA interventions [36]. Transferability of results from the LIFE study to this study is limited for several reasons. The LIFE study included an older population and did not assess time in MVPA or CRF, which has shown stronger associations to health outcomes in isotemporal substitution studies compared with LPA [37].

Meta-analysis data, including 12 RCTs, show that 3 aerobic/strength exercise sessions per week, for 30 to 60 min per session, can increase VO_2_max by approximately 2.3 mL/kg/min [25]. Furthermore, supervised exercise 3 times per week has shown to increase VO_2_max by approximately 6% over 12 weeks in adults [38]. This is somewhat comparable with the 2.5 mL/kg/min (7%) increase in VO_2_max seen in this study, with only 1 supervised 60-min aerobic/strength exercise session per week.

### Implications

Although the changes in PA were inconclusive, the changes in CRF (1.3 and 2.5 mL/min/kg increases in VO_2_max for the app group and supervised exercise group, respectively) may translate into substantial long-term health benefits. In fact, a 1 mL/min/kg increase in VO_2_max is associated with a 9% relative risk reduction of all-cause mortality (hazard ratio, 0.91; 95% CI 0.87 to 0.95) [39], which is a similar effect as a 10 cm reduction in waist circumference or a 10 mm/Hg reduction in systolic blood pressure [40]. Even though PA has effects on mortality independent of CRF, the opposite also holds true [41]. This further highlights the importance of the CRF improvements seen in this study. In addition, both protocols reduced waist circumference, fat mass, and BMI over 12 weeks, which are all important markers of health.

At baseline, participants in both groups had a slightly higher VO_2_max and were substantially more active (>80% of the participants meeting the PA recommendations) compared with the general Swedish population [42-44]. This may, to some extent, explain why participants in both groups did not increase PA over the intervention period. Moreover, as further discussed in the Strengths and Limitations section, the high levels of PA at baseline may limit generalizability of findings in this study to the general population with MD [7].

The app intervention was designed to be simple for widespread implementation in a variety of communities and settings, as it requires no special equipment or previous exercise knowledge. Although apps have the potential to increase the reach of health behavior change interventions, our results mirror the recent research showing that few individuals will use an offered app consistently over time [45]. Surprisingly, those who used the app ≥5 times/week did not show greater changes in any measured variables, from baseline to 12 weeks, compared with those who used the apps less frequently. Instead, the per-protocol analyses showed that those who used the apps more frequently had higher baseline levels of CRF and lower fat mass and BMI; thus, there was less room for improvements.

### Strengths and Limitations

A major strength of this study was the use of an RCT design to determine the effect of the use of commercially available apps compared with supervised exercise. Use of the apps during the intervention was ad libitum and not closely monitored, which reflects real-life app use, and contact with participants in the app group was minimal to reflect a real-world context and therefore increase generalizability. Furthermore, the primary and secondary outcomes were measured with objective measurements, in accordance with current recommendations for evaluating the effectiveness of PA interventions [36], which further adds to the study’s internal validity. Unlike the high attrition rate commonly observed in PA interventions, follow-up assessments were completed for 80% of participants, which represents a fairly high retention rate. As the study participants were aware of the study when they performed the baseline measurements of PA, it is most likely that they were more physically active than usual, which limits the comparison of baseline and outcome for PA measures. However, the CRF measures are reliable from baseline.

Some limitations of this study should be acknowledged. First, as participants were self-recruited from the community, they may not be fully representative of all people with MD, as shown by the high baseline levels of PA among the participants. Using free apps, instead of paid apps, may have increased contamination and/or cointerventions. There are also some limitations regarding our data collection. The participants began the intervention between December and May. Thus, changes in weather condition might have affected the levels of PA. However, there are data indicating that accelerometer-measured levels of PA may not be significantly affected by seasonality in populations living in high latitudes, such as Sweden [43]. In addition, we considered all valid MVPA observations from participants who provided 4 or more days of valid accelerometer data without considering the difference between weekdays and weekends. Both issues might introduce some bias in our analysis which, assuming successful randomization, would underestimate the effect of the intervention increasing the chance of type 2 error. Furthermore, the use of readily available apps precluded access to data on app utilization (we used self-reported data on app use).

The higher dropout in the app group (n=16), compared with the supervised exercise group (n=6), limited the statistical power to detect between-group differences. The initial power calculations were based on at least 40 participants, with measures on the primary outcome MVPA, in each group (n=39 in the app group at 12 weeks). However, 44% of the dropouts in the app group occurred directly after the randomization process because of dissatisfaction with the group allocation. Finally, the lack of a passive control group made it impossible to draw conclusions on whether apps can improve PA and CRF compared with not using apps. However, as stated in the aim of the study, the hypothesis of the study was to test to what extent commercially available apps can provide effects on PA and CRF compared with supervised exercise.

### Conclusions

Commercially available apps increased levels of CRF and improved body composition over 12 weeks to the same extent as supervised exercise sessions. However, neither the app-based intervention nor the supervised exercise intervention increased MVPA.

Given the high degree of smartphone use in the population, the fact that an app-based intervention has the potential to increase reach at a low cost and the substantial health effects associated with an increased CRF [12,39], this intervention may be an alternative approach to increase physical health–related outcomes in individuals with mild-to-moderate MD.

## Appendix

Multimedia Appendix 1 Tables, including appendix tables.

## Abbreviations

cpm: counts per minute
CRF: cardiorespiratory fitness
LMM: linear mixed-effect models
LPA: light physical activity
MD: mobility disability
MVPA: moderate-to-vigorous physical activity
PA: physical activity
RCT: randomized controlled trial
