# Supplementary material for: The Effect of Smartphone Apps Versus Supervised Exercise on Physical Activity, Cardiorespiratory Fitness, and Body Composition Among Individuals With Mild-to-Moderate Mobility Disability: Randomized Controlled Trial
Source: JMIR Mhealth Uhealth. 2020 Feb 4;8(2):e14615. doi: 10.2196/14615 (PMC7055745; doi:10.2196/14615)
Supplement: Multimedia Appendix 1 [file mhealth_v8i2e14615_app1.docx]

Table 1. Baseline characteristics of the participants by randomization group

| **Characteristic** | **App group (n=55) (SD)** | **Supervised exercise group (n=55) (SD)** |
| --- | --- | --- |
| Age (yrs) | 35.6 (6.2) | 34.5 (6.5) |
| Women (%) | 47 (87.0%) | 43 (78.2%) |
| *Smoking* | | |
| Daily smoking (%) | 3 (5.6%) | 2 (3.7%) |
| Smoking occasionally (%) | 11 (22.5%) | 5 (9.6%) |
| *Education* | | |
| Elementary school (%) | 3 (5.5%) | 2 (3.85%) |
| High school (%) | 12 (23.1%) | 14 (26.9%) |
| University (%) | 37 (67.3%) | 36 (65.5%) |
| *Moderate to vigorous physical activity (MVPA)* | | |
| MVPA (min/day) | 48.4 (23.3) | 40.3 (20.6) |
| *Cardiorespiratory fitness* | | |
| VO_2_ max (ml/kg/min) | 36.0 (7.9) | 35.3 (8.7) |
| *Body measures* |  |  |
| Weight (kg) | 77.6 (20.4) | 77.7 (17.3) |
| Height (cm) | 171.8 (9.4) | 171.0 (9.3) |
| BMI (kg/m^2^) | 26.3 (5.7) | 27.2 (5.2) |
| Fat mass (kg) | 27.1 (12.5) | 28.3 (11.9) |
| Fat-free mass (kg) | 49.1 (8.4) | 51.4 (10.6) |
| Waist circumference (cm) | 86.9 (18.3) | 84.6 (11.7) |

| **Time** | **MVPA (min/day)** | **VO_2_ max (ml/kg/min)** | **Weight (kg)** | **BMI (kg/m2)** | **Fat mass (kg)** | **Fat-free mass (kg)** | **Waist circumference (cm)** |
| --- | --- | --- | --- | --- | --- | --- | --- |
| **Baseline (95% CI)** | 5.72  (-1.48, 12.91) | 0.53  (-1.34, 2.40) | 2.17  (-0.42, 4.75) | -0.86  (-2.73, 1.02) | 0.86  (-0.28, 1.99) | 0.34  (-1.99, 2.67) | 4.27^**^  (0.76, 7.77) |
| **6 weeks (95% CI)** | -3.11  (-11.05, 4.84) | 0.86  (-1.09, 2.80) | 2.04  (-0.55, 4.64) | -0.79  (-2.67, 1.09) | 0.55  (-0.65, 1.75) | 1.18  (-1.23, 3.60) | 3.00  (-0.59, 6.59) |
| **12 weeks (95% CI)** | -1.82  (-9.76, 6.11) | -0.16  (-2.11, 1.80) | 2.16  (-0.44, 4.75) | -0.65  (-2.53, 1.23) | 0.13  (-1.08, 1.34) | 2.09  (-0.32, 4.51) | 4.28^*^  (0.69, 7.86) |

Table 2. Intention to treat analysis. Mean differences between the supervised exercise group (reference) and the App group over time for the primary and secondary outcomes

Adjusted for sex, BMI and VO_2_Max. ^*^ *P* < 0.05, ^**^ *P* < 0.01, ^***^ *P* < 0.001

Table 3. Intention to treat analysis. Pairwise comparison of the changes in primary and secondary outcomes at 6 weeks and 12 weeks by randomization group

| **App group (n=55)** | | |
| --- | --- | --- |
| **Characteristic** | Δ **0 to 6 weeks (95% CI)** | Δ **0 to 12 weeks (95% CI)** |
| *Moderate to vigorous physical activity (MVPA)* | | |
| MVPA (min/day) | -2.25 (-10.40, 5.90) | -6.16 (-14.28, 1.95) |
| *Cardiorespiratory fitness* | | |
| VO_2_ max (ml/kg/min) | 1.32 (0.13, 2.52)^**^ | 1.07 (-0.14, 2.27)^*^ |
| *Body measures* | | |
| Weight (kg) | 0.16 (-0.35, 0.66) | -.03 (-0.53, 0.47) |
| BMI (kg/m^2^) | -0.09 (-0.43, 0.26) | -0.19 (-0.54, 0.16) |
| Fat mass (kg) | -0.01 (-0.89, 0.92) | 0.20 (-0.73, 1.12) |
| Fat-free mass (kg) | 0.77 (-0.65, 2.19) | 0.52 (-0.93, 1.96) |
| Waist circumference (cm) | -0.16 (-1.90, 1.57) | -0.75 (-2.49, 0.99) |
| **Supervised exercise group (n=55)** | | |
| **Characteristic** | Δ **0 to 6 weeks (95% CI)** | Δ **0 to 12 weeks (95% CI)** |
| *Moderate to vigorous physical activity (MVPA)* | | |
| MVPA (min/day) | 6.57 (-0.74, 13.89)^*^ | 1.37 ( -6.05, 8.81) |
| *Cardiorespiratory fitness* | | |
| VO_2_ max (ml/kg/min) | 0.99 (-0.07, 2.07)^*^ | 1.77 (0.70, 2.83)^***^ |
| *Body measures* | | |
| Weight (kg) | 0.28 (-0.17, 0.72) | -0.02 (-0.49, 0.45) |
| BMI (kg/m^2^) | -0.16 (-0.46, 0.15) | -0.40 (-0.72, -0.07)^**^ |
| Fat mass (kg) | 0.32 (-0.50, 1.13) | 0.92 (0.09, 1.75)^**^ |
| Fat-free mass kg) | -0.08 (-1.35, 1.20) | -1.24 (-2.54, 0.06)^*^ |
| Waist circumference (cm) | 1.10 (-0.44, 2.64) | -0.76 (-2.34, 0.82) |

^*^ *P* < 0.05, ^**^ *P* < 0.01, ^***^ *P* < 0.001

**Appendix Tables**

| **Time** | **MVPA (min/day)** | **VO_2_ max (ml/kg/min)** | **Weight (kg)** | **BMI (kg/m2)** | **Fat mass (kg)** | **Fat-free mass (kg)** | **Waist circumference (cm)** |
| --- | --- | --- | --- | --- | --- | --- | --- |
| **Baseline (95% CI)** | 8.80  (-1.99, 19.60) | 3.32^*^  (0.54, 6.10) | 0.34  (-2.81, 3.49) | -4.54^***^  (-6.94, -2.15) | 0.25  (-1.45, 1.95) | 0.59  (-2.94, 4.13) | 0.65  (-1.58, 2.89) |
| **6 weeks (95% CI)** | -5.83  (-17.07, 5.40) | 3.76^**^  (-0.98, 6.55) | 0.36  (-2.78, 3.50) | -4.24^**^  (-6.64, -1.84) | 0.31  (-1.41, 2.03) | 1.50  (-2.06, 5.06) | 0.47  (-1.82, 2.75) |
| **12 weeks (95% CI)** | -4.45  (-15.67, 6.77) | 2.51  (-0.28, 5.29) | -0.01  (-3.13, 3.10) | -4.06^**^  (-6.45, 1.67) | -0.95  (-2.67, 0.77) | 2.76  (-0.78, 6.30) | 1.43  (-0.84, 3.70) |

Table 1a. Per-protocol analysis. Mean differences between the supervised exercise group (reference) and the App group over time for the primary and secondary outcomes.

Adjusted for sex, BMI and VO_2_Max. ^*^ *P* < 0.05, ^**^ *P* < 0.01, ^***^ *P* < 0.001

Table 2a. Per-protocol analysis. Pairwise comparison of the changes in primary and secondary outcomes at 6 weeks and 12 weeks by randomization group

| **App group (n=20)** | | |
| --- | --- | --- |
| **Characteristic** | Δ **0 to 6 weeks (95% CI)** | Δ **0 to 12 weeks (95% CI)** |
| *Moderate to vigorous physical activity (MVPA)* | | |
| MVPA (min/day) | -8.49 (-20.65, 3.68) | -11.82 (-24.23, 0.58)^*^ |
| *Cardiorespiratory fitness* | | |
| VO_2_ max (ml/kg/min) | 1.34 (-0.55, 3.22) | 1.17 (-0.77, 3.11) |
| *Body measures* | | |
| Weight (kg) | 0.25 (-0.55, 1.05) | -.40 (-1.23, 0.42) |
| BMI (kg/m^2^) | -0.08 (-0.48, 0.63) | 0.02 (-0.55, 0.59) |
| Fat mass (kg) | 0.36 (-0.98, 1.70) | -0.23 (-1.64, 1.17) |
| Fat-free mass (kg) | 0.78 (-1.55, 3.12) | 0.93 (-1.52, 3.37) |
| Waist circumference (cm) | 0.34 (-1.96, 2.38) | -0.49 (-2.59, 1.60) |
| **Supervised exercise group (n=45)** | | |
| **Characteristic** | Δ **0 to 6 weeks (95% CI)** | Δ **0 to 12 weeks (95% CI)** |
| *Moderate to vigorous physical activity (MVPA)* | | |
| MVPA (min/day) | 6.15 (-1.63, 13.94)^*^ | 1.43 ( -6.57, 9.44) |
| *Cardiorespiratory fitness* | | |
| VO_2_ max (ml/kg/min) | 0.89 (-0.31, 2.19) | 1.98 (0.77, 3.19)^***^ |
| *Body measures* | | |
| Weight (kg) | 0.23 (-0.29, 0.75) | -0.05 (-0.61, 0.52) |
| BMI (kg/m^2^) | -0.23 (-0.59, 0.13) | -0.46 (-0.85, -0.08)^**^ |
| Fat mass (kg) | 0.29 (-0.57, 1.16) | 0.96 (0.07, 1.86)^**^ |
| Fat-free mass kg) | -0.12 (-1.64, 1.34) | -1.24 (-2.82, 0.34)^*^ |
| Waist circumference (cm) | 0.53 (-1.69, 2.38) | -1.27 (-2.60, 0.07)^*^ |

^*^ *P* < 0.05, ^**^ *P* < 0.01, ^***^ *P* < 0.001

Table 3a. Between group differences at 6 and 12 weeks in the primary outcome and secondary outcomes, including participants with complete data at 12 weeks.

|  | **6 weeks** | | | **12 weeks** | | |
| --- | --- | --- | --- | --- | --- | --- |
| **Characteristic** | **App group (n=39) (SD)** | **Supervised exercise (n=49) (SD)** | **P-value** | **App group (n=39) (SD)** | **Supervised exercise (n=49) (SD)** | **P-value** |
| *Moderate to vigorous physical activity (MVPA)* | | | | | | |
| MVPA (min/day) | 47.0 (14.4) | 49.3 (18.1) | 0.51 | 43.6 (21.9) | 44.9 (19.0) | 0.76 |
| *Cardiorespiratory fitness* | | | | | | |
| VO_2_ max (ml/kg/min) | 38.4 (7.7) | 36.5 (8.3) | 0.28 | 38.1 (7.9) | 37.6 (9.4) | 0.80 |
| *Body measures* | | | | | | |
| Weight (kg) | 75.5 (15.5) | 79.7 (15.7) | 0.21 | 77.5 (20.3) | 78.4 (15.7) | 0.81 |
| BMI (kg/m^2^) | 25.4 (4.9) | 27.1 (5.2) | 0.11 | 25.6 (5.1) | 26.8 (5.3) | 0.27 |
| Fat mass (kg) | 25.2 (11.4) | 28.2 (11.6) | 0.22 | 25.3 (11.5) | 27.8 (12.2) | 0.35 |
| Fat-free mass (kg) | 50.3 (8.8) | 51.4 (10.4) | 0.61 | 50.3 (8.9) | 50.1 (11.5) | 0.95 |
| Waist circumference (cm) | 83.4 (14.5) | 85.4 (11.0) | 0.45 | 82.5 (14.0) | 82.7 (10.4) | 0.94 |

Table 4a. Baseline characteristics of participants lost to follow-up by randomization group

| **Characteristic** | **App group (n=16)** | **Supervised exercise group (n=6)** | ***P*-value** |
| --- | --- | --- | --- |
| Female | 16 (94%) | 5 (83%) | 0.46 |
| Age | 36.5 (6.60) | 37.2 (8.45) | 0.85 |
| *Moderate to vigorous physical activity (MVPA)* | | | |
| MVPA (min/day) | 49.2 (25.7) | 29.2 (17.5) | 0.094 |
| *Cardiorespiratory fitness* | | | |
| VO_2_ max (ml/kg/min) | 34.3 (7.2) | 35.4 (12.2) | 0.80 |
| *Body measures* | | | |
| Weight (kg) | 75.2 (19.3) | 74.5 (10.5) | 0.93 |
| Height (cm) | 167.7 (6.3) | 168.5 (9.9) | 0.83 |
| Waist circumference (cm) | 89.8 (24.4) | 82.9 (8.6) | 0.51 |
| BMI (kg/m^2^) | 26.8 (6.6) | 26.3 (3.7) | 0.86 |
| Fat mass (kg) | 29.0 (14.5) | 26.7 (9.5) | 0.72 |
| Fat free mass (kg) | 46.7 (6.9) | 47.8 (7.4) | 0.75 |

Table 5a. Baseline characteristics by participants and drop-outs

| **Characteristic** | **Participants (n=88)** | **Drop-outs (n=22)** | ***P*-value** |
| --- | --- | --- | --- |
| Female | 68 (80%) | 21 (91%) | 0.35 |
| Age | 34.6 (6.2) | 36.7 (6.9) | 0.16 |
| *Moderate to vigorous physical activity (MVPA)* | | | |
| MVPA (min/day) | 44.9 (21.3) | 44.0 (25.0) | 0.86 |
| *Cardiorespiratory fitness* | | | |
| VO_2_max (ml/kg/min) | 36.0 (8.3) | 34.6 (8.2) | 0.48 |
| *Body measures* | | | |
| Weight (kg) | 78.4 (19.2) | 75.0 (17.2) | 0.45 |
| Height (cm) | 172. (9.6) | 167.9 (7.1) | 0.043 |
| Waist circumference (cm) | 84.5 (13.3) | 88.0 (21.4) | 0.34 |
| BMI (kg/m^2^) | 26.7 (5.4) | 26.7 (5.9) | 0.97 |
| Fat mass (kg) | 27.5 (11.9) | 28.4 (13.2) | 0.75 |
| Fat Free mass (kg) | 51.2 (10.0) | 47.0 (6.9) | 0.065 |
